# BranchClust: a phylogenetic algorithm for selecting gene families

**DOI:** 10.1186/1471-2105-8-120

**Published:** 2007-04-10

**Authors:** Maria S Poptsova, J Peter Gogarten

**Affiliations:** 1 Department of Molecular and Cell Biology, University of Connecticut, Storrs, CT 06269-3125, USA

## Abstract

**Background:**

Automated methods for assembling families of orthologous genes include those based on sequence similarity scores and those based on phylogenetic approaches. The first are easy to automate but usually they do not distinguish between paralogs and orthologs or have restriction on the number of taxa. Phylogenetic methods often are based on reconciliation of a gene tree with a known rooted species tree; a limitation of this approach, especially in case of prokaryotes, is that the species tree is often unknown, and that from the analyses of single gene families the branching order between related organisms frequently is unresolved.

**Results:**

Here we describe an algorithm for the automated selection of orthologous genes that recognizes orthologous genes from different species in a phylogenetic tree for any number of taxa. The algorithm is capable of distinguishing complete (containing all taxa) and incomplete (not containing all taxa) families and recognizes in- and outparalogs. The BranchClust algorithm is implemented in Perl with the use of the BioPerl module for parsing trees and is freely available at .

**Conclusion:**

BranchClust outperforms the Reciprocal Best Blast hit method in selecting more sets of putatively orthologous genes. In the test cases examined, the correctness of the selected families and of the identified in- and outparalogs was confirmed by inspection of the pertinent phylogenetic trees.

## Background

The problem of gene family selection from any given set of taxa is one of the primary tasks in evolutionary genomics. The correct identification of orthologs (i.e. genes whose deepest relationship represents a speciation event [1]) is crucial for reconstruction and interpretation of phylogenetic trees; and for addressing the following questions: How many common genes are shared by different species? What is the extent of the core of genes that shares a common history? Which genes underwent duplication, were lost, or horizontally transferred between different lineages? Most of the known methods used for detection of orthologs are based on sequence similarity and genome-specific best hits [2]. Phylogenetic methods are more reliable but they are difficult to automate and the complexity grows with the increase of the number of taxa in question.

Another important task in molecular evolution is to ascribe a gene function to open reading frames in a newly sequenced genome. Today's gene annotation techniques are based largely on a search for homologous sequences with known functions, with orthologs more likely to have identical functions as compared to paralogs. The methods are based on sequence similarity searches using BLAST [3] as a primary tool, and PSI-BLAST [4] or HMM-based methods for profile searching [5]. The final stage of positioning of an unknown sequence in a phylogenetic tree in order to infer its function either needs to be performed manually by the curator, or is omitted entirely, which can be problematic because changes in substitution rate frequently lead to situations where the closest phylogenetic neighbor does not correspond to the highest scoring hit in a BLAST search [6]. The algorithm we propose here fully automates the process of assembling gene families for any given number of taxa and also aids sequence annotation because it positions an unknown gene sequence in a phylogenetic tree containing both paralogs and orthologs from closely related species.

**Figure 1:**
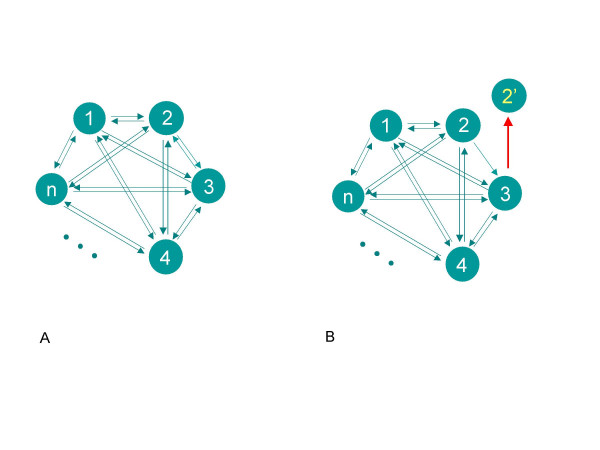
**The reciprocal best BLAST hit method**. Circles represent genes from n different taxa, arrows signify best BLAST hit relationship; (A) – case of strict reciprocity, (B) – failing of reciprocity in the presence of paralogs.

Today, a widely used method to identify sets of orthologs from a set of *n *species is the reciprocal best BLAST hit method (e.g., [7,8]). The method requires strong conservative relationships among the orthologs so that if a gene from species 1 selects a gene from species 2 as a best hit when performing a BLAST search with genome 1 against genome 2, then the gene 2 must in turn select gene 1 as the best hit when genome 2 is searched against genome 1. For a set of *n *species the reciprocal BLAST hit method requires the presence of all pairwise reciprocal connections between all species as depicted on Figure 1A.

The reciprocal BLAST hit method is very stringent and succeeds in the selection of conserved orthologs with a low false positive rate [9], but it often fails to assemble sets of orthologs in the presence of paralogs. Figure 1B illustrates how reciprocity is broken in the presence of paralogous gene 2' closely related to gene 2. Genes 2 and 2' could be inparalogs [10] that resulted from a recent gene duplication. In this example gene 3 chooses gene 2' instead of gene 2 as a best BLAST hit, preventing both paralogs from being appropriately recognized as orthologs [1].

**Figure 2:**
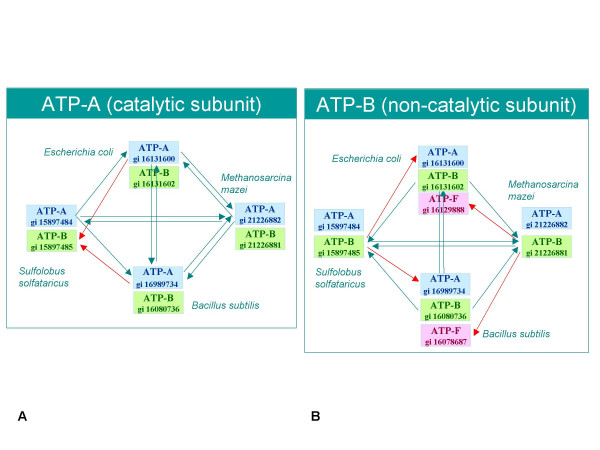
**Diagram showing best BLAST hit relationships between ATP synthases, subunit A (Figure 2A) and subunit B (Figure 2B) for two bacteria, *Escherichia coli *and *Bacillus subtilis*, and two archaea, *Methanosarcina mazei *and *Sulfolobus solfataricus***. Some confusion prevails in the annotation of ATP synthase subunits for bacteria and archaea: the beta chain in bacteria is the catalytic subunit and corresponds to subunit A in archaeal and eukaryotic vacuolar type ATPases; the alpha chain, or non-catalytic subunit, in bacteria corresponds to subunit B in archaea [46]. In addition, the archaeal A subunit is sometimes labelled as alpha subunit. To simplify our discussion and the diagrammatic representation we designate all catalytic subunits, either from bacteria or from archaea as ATP-A, and all non-catalytic subunits as ATP-B.

**Figure 3:**
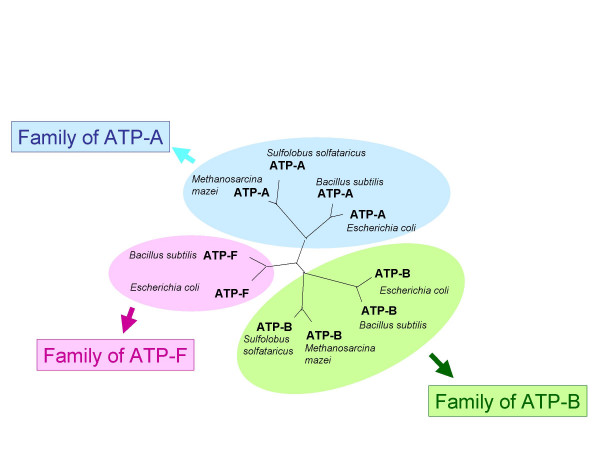
**Phylogenetic tree reconstructed for a superfamily of ATP synthase subunits**. The tree was reconstructed by distance method using ClustalW [41]. Maximum likelihood based reconstruction using Phyml [43] with JTT model results in the same tree topology.

Figure 2 gives an example where reciprocity fails in a case of four species (two archaea and two bacteria) for a conserved anciently duplicated protein, ATP synthase ATP binding subunits. In the case of the catalytic subunits (ATP-A, Figure 2A), reciprocity is broken when ATP-A from *Escherichia coli *and *Bacillus subtilis *chooses the more conserved subunits B (ATP-B), from *Sulfolobus solfataricus *as the best BLAST hit. In the case of the ATP-B family (Figure 2B), the situation is further complicated by the presence of a third paralog frequently found in bacterial species, a paralog that is involved in assembly of the bacterial flagella [11] (here denoted as ATP-F), which is selected as the best hit for ATP-B from the archaeon *Methanosarcina mazei*. As a result, neither ATP-A nor ATP-B are selected as gene families when applying a strict reciprocal best BLAST hit method. In many bacteria additional ATP-A paralogs exist that make the recognition of orthologs even more difficult (see below): a Rho transcription termination factor involved in unwinding the RNA transcript from the encoding DNA, and an ATPase that is part of type III secretion systems that is similar to ATP-F. In contrast to the reciprocal best hit approach, a phylogenetic tree, reconstructed for all the genes collected from both diagrams of Figure 2, places ATP-A, ATP-B and ATP-F on separate branches forming three distinct clusters representing the three gene families (see Figure 3).

In total, from the four species mentioned above, the strict reciprocal best BLAST hit method selects only 80 families. To illustrate the influence of paralogs on the total number of selected orthologs, we conducted the following experiment. Each genome was "BLASTed" against itself, and if a query gene had significant hits other than the query itself, these genes were removed from further consideration. Then the reciprocal best BLAST hit method was applied to the genomes "cleaned" from paralogs, and the number of selected families increased to 200, demonstrating that the small size reported for the prokaryotic core (see [12] for examples) is in part due to difficulties discriminating between in- and outparalogs. Because the genomes were artificially reduced by removing all paralogs (including out paralogs) that could cause confusion, the total number of orthologous families for complete genomes should be even bigger.

In the Clusters of Orthologous Groups (COG) [13] strict reciprocity is replaced by a triangular best Blast hits relationship. First, triangles forming one-side circular best BLAST hits are constructed, and then triangles with common sides are merged together to form a cluster. The COG clusters consist of an undifferentiated mixture of orthologs and in- and outparalogs, and are limited to a certain set of taxa. The same problem exists for HOBACGEN [14] whose precomputed database of gene families also is a mixture of orthologs and paralogs and does not contain many of the newly sequenced genomes.

A simple phylogenetic algorithm to infer speciation and duplication events in a gene tree was proposed by [15] but their approach requires a known species tree which is impossible in many cases of prokaryotes. Another limitation of this method is that all incongruence is explained by means of duplication and losses, whereas for prokaryotes, these often, result from HGT events.

The Inparanoid euykaryotic orthologs database [16] contains pairwise orthologs which were assembled by the reciprocal best BLAST hit method. Inparalogs are added to the orthologous pairs by applying the Inparanoid clustering method based on sequence similarities scores [17]. Only two taxa are considered at a time.

We aimed to develop a method of assembling orthologous gene families that would have no restriction on the number of taxa, doesn't require a known species tree and would be able to distinguish between paralogs and orthologs by analyzing their position in a phylogenetic tree.

## Results

### Algorithm

In molecular evolution the notion of branch is frequently used as synonymous for a split, i.e. the line connecting two nodes. Throughout the article we use the term branch in its traditional meaning, as referring to a subtree in a rooted tree that contains all nodes distal to a node. In the following, the term genome refers to the collection of all amino acid sequences encoded in a genome, and with genome BLAST we denote the collection of all BLAST searches, performed with blastall [3], using each of the amino acid sequences as query. We use the term cluster to denote a subtree containing one set of orthologous genes. If there is more than one gene from the same taxon inside a cluster, these genes are considered as inparalogs. We use the term superfamily to denote a collection of genes that show significant similarity to each other.

Here we present BranchClust, a branch clustering algorithm that parses trees to delineate families of orthologs within a superfamily containing several paralogous gene families. The underlying idea is that closely related genes are placed on one branch emerging from one node on a tree, so the task of detecting families for *n *different taxa is simply a task to detect branches containing groups of genes from all, or almost all, species. Step-by-step guidance on all procedures of the method from downloading complete genomes to applying the BranchClust algorithm and using BranchClust in conjunction with TreeDyn [18] are available in the BranchClust tutorial at [19].

First, we start with selection of the so-called superfamilies, i.e. sets of genes containing mixtures of orthologs and paralogs. Assume that we have *n *complete genomes of different taxa. We combine all genomes from *n *taxa in one database. From this set of *n *genomes, we arbitrarily choose one and perform BLAST searches of all genes in the selected genome against a combined genome database. Then we parse the BLAST output in such a way that not only the best hit for each species is selected, but all of the significant hits obtained for a given query. As a result, each species can contribute to a superfamily through both orthologs and paralogs of the original query. Superfamilies constructed from the paralogs of the query species are combined in one.

The choice of a starting genome could affect the ultimate number and composition of families of orthologous genes, especially, if we relax the criteria and require that at least 80% of different taxa be present in each family. Different species might collect different sets of significant hits. To select all possible orthologs we perform BLAST of all *n *genomes against the database composed of the same *n *genomes, and then assemble all significant hits for each gene from every genome in superfamilies (see the BranchClust Tutorial [19] for a step by step description for assembling superfamilies).

The sequences from obtained superfamilies are aligned and a phylogenetic tree is reconstructed by any of the preferred methods of tree reconstruction (currently by default clustalw 1.83 is used for sequence alignment with default parameters and for phylogenetic reconstruction using correction for multiple substitutions; see Methods and Discussion for more details).

DEFINITION 1. We will call a branch in a phylogenetic tree complete if it contains representatives from all species, and incomplete otherwise.

DEFINITION 2. We will call a node in a phylogenetic tree complete if a complete branch originates from that node, and incomplete otherwise.

**Figure 4:**
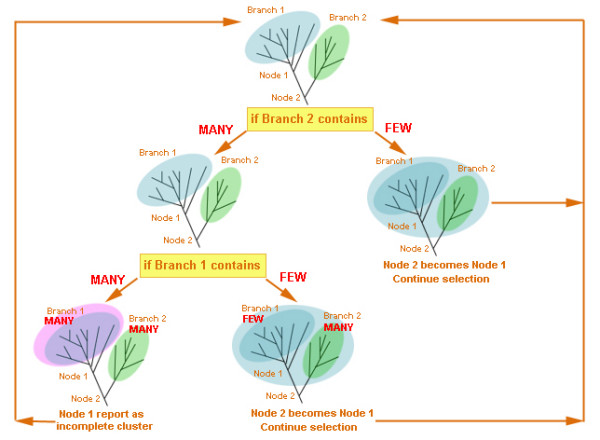
Schematic representation of the BranchClust algorithm.

We start the selection from the most distant leaf in a tree (the choice of the root will be discussed later), and then descend node by node, adding branches and calculating the total number of different species on the branches, until a node becomes complete. A schematic representation of BranchClust algorithm is depicted in Figure 4.

We start from Node 1 which contains an incomplete Branch 1. Then we descend to Node 2 and add Branch 2 containing some number of different taxa. Here we introduce the notion of "FEW" and "MANY". Depending on the number of considered species, parameter "MANY" signifies how many different taxa would be sufficient for a branch to be reported as a separate cluster, or, in other words, the parameters "MANY" and FEW" determine which branch could serve as a "stopper" when it is encountered as a side branch while descending from node to node (see Figure 4). The boundary between FEW and MANY can be adjusted (for example in cases where many reduced genomes are included); usually a boundary between "FEW" and "MANY" around 70–80% of the different taxa in question was found to work satisfactorily. For example, in case of 4 taxa, "MANY" would be more than 2 or 3; in case of 13 taxa – 8–10, and in case of 30 – 20–25. "FEW" is just the total number of different taxa minus "MANY". If Branch 2 contains "FEW" species we continue selection until Node 2 becomes complete. If Branch 2 contains "MANY" species, then we must check how many species are already on Branch 1. If Branch 1 contains "MANY" species, and we also added "MANY" species on Branch 2, then it is most likely that Branch 1 is a separate, though incomplete, cluster. If Branch 1 contains only "FEW" different species, then we add it to the Branch 2, and continue with the selection.

The algorithm's pseudocode can be written as follows:

INPUT:

   Binary superfamily tree T for n taxa.

OUTPUT:

   Clusters, either complete or incomplete, with report of selected families, inparalogs and out-of-cluster paralogs.

NEW SELECTION:

   while (Tree T has leaves):

INITIATION:

Find the leaf most distant from the current, arbitrarily selected root and set Node 1 as the ancestor of the most distant leaf.

RECURSION:

   check if the ancestor of the Node1 has a "stopper", leaf "R", that signifies previously removed cluster.

   if (ancestor of the Node 1 has leaf "R") ->

         Branch 1 contains a cluster, report an incomplete cluster, remove it, mark the Node 1 with a leaf "R", re-root the tree with cluster's ancestor and go to NEW SELECTION:

   calculate number of different taxa on the Branch 1: n1

   calculate number of different taxa on the Branch 2: n2

   calculate total number of different taxa on the Node 2: n3

   if (n1 > = n) ->

      Node 1 is complete, report a complete cluster, remove it from the tree, mark the Node 1 with a leaf "R", re-root the tree with cluster's ancestor and go to NEW SELECTION:

   else – Node 1 is incomplete, check the state of the Node 2

      case (Branch 1, Branch 2) contains combinations:

         ((FEW, FEW), (FEW, MANY), (MANY, FEW)) and (n3 < n) -> Node 2 becomes Node 1, go to RECURSION.

         ((FEW, FEW), (FEW, MANY), (MANY, FEW)) and (n3 > = n) -> Node 2 is complete, report a complete cluster, remove it, mark the Node 2 with a leaf "R", re-root the tree with cluster's ancestor and go to NEW SELECTION:

         (MANY, MANY) -> Branch 1 contains a cluster, report an incomplete cluster, remove it, mark the Node 1 with a leaf "R", re-root the tree with cluster's ancestor and go to NEW SELECTION:

END OF RECURSION

**Figure 5:**
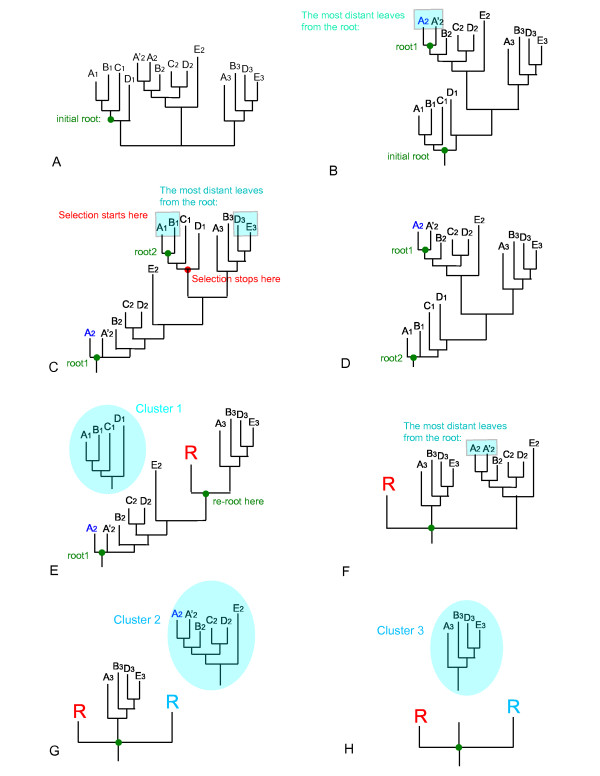
**Example of BranchClust selection steps for a superfamily tree for 5 different taxa with 3 clusters**. Figure 5A. Unrooted original tree. Figure 5B. Tree is initially rooted inside the cluster. The ancestor of the most distant leaf from the root is set to be root 1. Figure 5C. Tree is re-rooted with the root 1. The ancestor of the most distant leaf from the root 1 is set to be root 2. Selection starts from the most distant from the root 1 leaf and ends when it encounters branch containing MANY (here 4) species. First cluster is selected and removed from the tree. The node is marked with a leaf "R". Figure 5D. Tree is re-rooted with the root 2. Figure 5E. Tree is re-rooted at the ancestor of the selected cluster and the selection continues. Figure 5F. Cluster 2 is selected, removed from the tree, the node of cut is marked with a leaf "R". Figure 5G. Tree is not re-rooted because the ancestor of the removed cluster is already the root. Selection continues. The last cluster is selected. End of selection.

When a tree containing several clusters (i.e., families of orthologs) is submitted to the BranchClust algorithm, it is arbitrarily rooted: it can be rooted inside any cluster or anywhere in between (see example below). For example, if the root splits a cluster so that one branch will contain more than MANY species, this branch will be wrongly reported as a family. However, if a tree is rooted somewhere in between the clusters, the results will not be affected by the root position because a branch with a cluster acts as a "stopper". To avoid artifacts due to placing the initial root, the selection is repeated for the tree rooted at the opposite end. We report as final the clustering that minimizes the number of paralogs. The process of selection with tree re-rooting is illustrated in Figure 5.

Figure 5A shows a hypothetical unrooted tree for a set of 5 taxa A, B, C, D and E. The parameter MANY is set to 4 (i.e. the branch containing 4 different taxa will serve as a "stopper"). The algorithm runs twice with two different roots, which are chosen as the two nodes most distant from each other. The process of root selection for the two independent runs is shown on Figures 5B–D. Figures 5C, E–H show how BranchClust works for the tree rooted with root 1.

**Figure 6:**
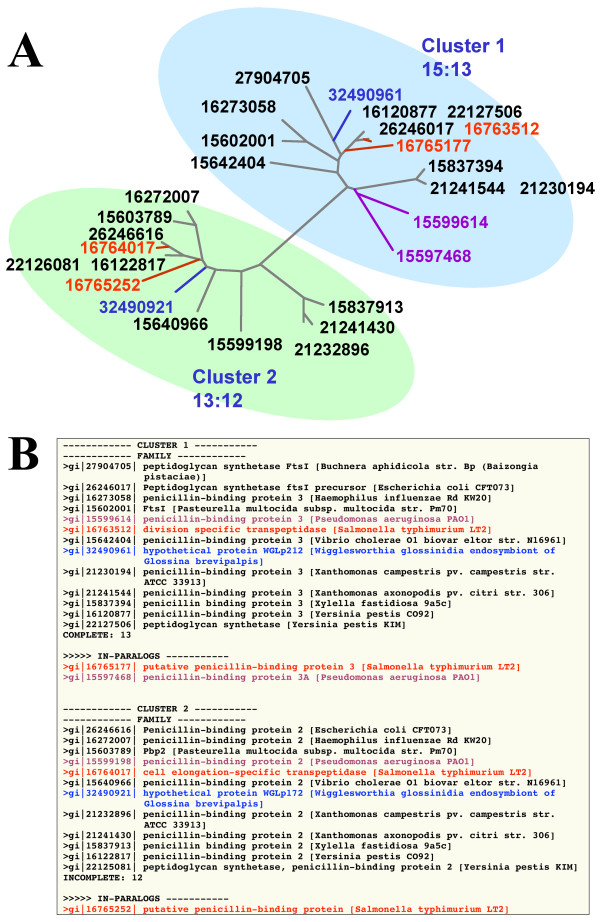
**Superfamily of penicillin-binding proteins for 13 gamma proteobacteria**. Clustering performed with MANY = 8. In *Salmonella typhimurium *there are two paralogs, gi 16763512 and gi 16765177, in Cluster 1; and two paralogs, gi 16765252 and gi 16764017 in Cluster 2 (red color); and there are two paralogs of *Pseudomonas aeruginosa*, gi 15597468 and gi 15599614 in Cluster 1 (violet color). Two proteins labeled as hypothetical in the genome annotation are highlighted in blue. n:m indicates that the highlighted cluster contains n leaves from m different taxa. See Methods for the list of taxa. Panel A depicts the phylogeny reconstructed form the sequences, panel B gives the ouput of the BranchClust algorithm.

We repeat selection for the tree rooted with root 2 (Figure 5D) and compare the results by calculating the number of paralogs resulting in two different runs. The clustering that contains the least number of paralogs is selected. Using two trees rooted at opposing ends helps to solve a problem that arises in case of two incomplete clusters. This problem and how it is addressed by the implemented approach is illustrated by the clustering of the penicillin binding proteins' superfamily for a set of 13 gamma proteobacteria (Figure 6A).

The superfamily containing the penicillin-binding proteins consists of 25 members that form two distinct clusters in the tree: one is a branch with 15 leaves and 13 different taxa, forming a complete cluster; the other cluster is incomplete, containing only 12 members from 12 different species. The results of BranchClust algorithm in this case depend on the starting point, or the root of the tree. If we start selection inside Cluster 1, we will select the complete Cluster 1, remove it from the tree and the remaining tree will be the incomplete Cluster 2. However, if we start selection inside Cluster 2, we will skip the node containing Cluster 2 and continue selection to form a complete branch. This will result in the following clustering: 23:13, 5:5, meaning that one branch contains 23 leaves with 13 different taxa and the second – 5 leaves with 5 different taxa. The number of paralogs is given by the difference between the number of leaves on a branch and number of different taxa. In the latter case this number would be 23-13 + 5-5 = 10, and in the first run we have the difference of 15-13+13-12 = 3. We select that run which yields the minimum number of paralogs.

Once a cluster is isolated, a family containing one representative from each taxon is selected with identification of inparalogs as duplicated genes inside that cluster. For example, in the case of penicillin-binding proteins' superfamily (Figure 6A–B), cluster 1 contains two inparalogs, one of *Salmonella typhimurium*, gi 16765177, and the other is of *Pseudomonas aeruginosa*, gi 15597468. Selection starts from the most distant leaf on a tree, and the gene copy which is closest to the top of the branch is reported as part of the family, while other copies are reported as inparalogs.

**Figure 7:**
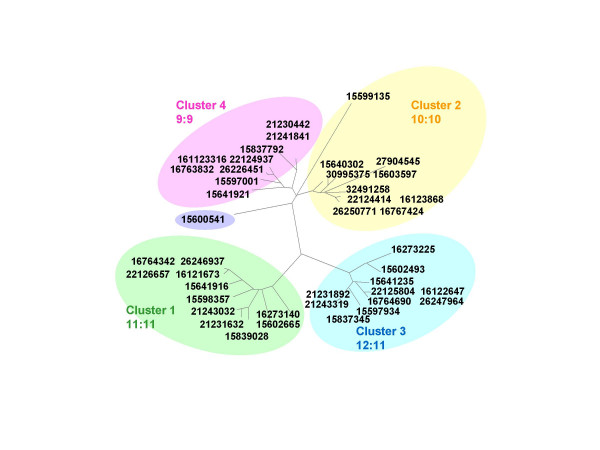
**Superfamily of DNA-binding proteins and Integration host factors for 13 gamma proteobacteria**. Clustering performed with MANY = 8. There are four incomplete clusters and one additional out-of-cluster paralog of *Pseudomonas aeruginosa*, gi 15600541.

We call genes "out-of-cluster paralogs", if they are located inside a superfamily, but not on the branch containing a selected cluster. Note that for a given cluster all other genes from the same superfamily are "outparalogs". We do not include all of these outparalogs in our clustering reports, because this would just list all genes in the superfamily not included in that cluster. The concept of "out-of-cluster paralogs" is illustrated in Figure 7, which depicts the superfamily of DNA-binding proteins and integration host factors for 13 gamma proteobacteria. The second copy of gene from *Pseudomonas aeruginosa*, gi 15600541, is reported as an out-of-cluster paralog (Figure 7) because *Pseudomonas *contains one copy inside each cluster. See [19] and additional files for more examples.

### Testing

#### Clustering

We tested the BranchClust algorithm on four different sets of genomes: 2 bacteria and archaea, 13 gamma proteobacteria, 14 archaea and 30 bacteria and archaea together. Table 1 compares the number of families of orthologs selected by the reciprocal best BLAST hit method and by the BranchClust algorithm.

**Table 1:** Comparison of the best BLAST hit method and BranchClust algorithm.

**Number of taxa – A: Archaea B: Bacteria**	**Number of selected families:**	
	
	**Reciprocal best BLAST hit**	**BranchClust**
2A2B	80	414 (all complete)
13B	236	2066 (369 complete, 1690 with n ≥ 8)
14A	125	1431 (300 complete, 1131 with n ≥ 8)
14B 16A	12	195 (80 complete, 195 with n ≥ 24)

The homologs of ATPase/ATPsynthase catalytic subunits provide a good test case to explore the limits of algorithms to assemble families of orthologs. This superfamily includes ancient paralogs and recent gene duplications, and among the homologs that are part of the type three secretions system are genes frequently horizontally transferred found in pathogenicity islands [20,21]. Examples of clustering the ATP synthases' superfamily for 13 gamma proteobacteria, 30 and 317 bacteria and archaea can be found in the additional files [see Additional files 1, 2, 3] and on the BranchClust web-site [19]. In all cases BranchClust recognizes complete clusters for ATP-A and ATP-B, as well as clusters for Rho-termination factor, and ATP-F and type III secretion system ATPases. More examples of BranchClust analyses are given in the additional files: examples from the analysis of 13 gamma proteobacterial genomes [see Additional files 4, 5, 6, 7, 8]; analysis of 14 archaeal genomes [see Additional files 9, 10, 11, 12, 13]; and for a joint analysis of 16 bacterial and 14 archaeal genomes [see Additional file 14, 15, 16, 17, 18].

#### Gene annotation

An example of how the BranchClust algorithm could aid gene annotation is provided by the example of the penicillin-binding proteins superfamily (Figure 6A). The current annotation for clusters reported by the BranchClust algorithm is presented in Figure 6B. It is evident from the report that the two hypothetical proteins of *Wigglesworthia glossinidia *are likely to be penicillin-binding proteins 2 and 3, respectively. In the case of paralogs of *Salmonella typhimurium*, the annotation is confusing. The nearest neighbors of *Escherichia coli *are annotated as division-specific transpeptidases (gi 16763512 and gi 16764017), and the paralogs, more distant to *Escherichia coli*, are identified as penicillin-binding proteins (gi 16765177 and gi 16765252).

Moreover, genes within each cluster are not consistently named. For example, 7 genes out of 13 are annotated as penicillin-binding protein 3, and other members of the family as peptidoglycan synthetase ftsI, or its precursor (*Escherichia coli*, *Buchnera aphidicola*, *Pasteurella multocida *and *Yersinia pestis*). Only specialists would recognize that penicillin-binding protein 3 and peptidoglycan synthetase ftsI are synonymous and designate the same protein. Perhaps more species and additional structural analysis will be needed to infer the correct gene names, but the BranchClust algorithm can be used as a first automated step in the complex task of gene annotation. See additional files for more examples of annotations inside clusters.

### Implementation

The BranchClust algorithm is implemented in Perl with the use of BioPerl module for parsing trees and is available at [19].

### Automation with TreeDyn

The best way to evaluate the results of clustering is to highlight the selected clusters, families, and in- and outparalogs in a phylogenetic tree. Graphic representation of a clustered tree can be automated using the TreeDyn program [18]. An example of how the results of the BranchClust are highlighted for a tree of ATP-ases' superfamily for 13 gamma proteobacteria together with labelling file for the automated use of TreeDyn are available in the BranchClust Tutorial.

## Discussion

### Gene family, family of orthologs and superfamily

At present there is no agreement on the definitions of the terms gene family and superfamily. Usually the term gene family implies homology, i.e. descent from a common ancestral gene, but this is a very sparse definition, because there is no indication of how far back in time a common origin should be traced. We used orthology as criterion defining clusters and included the collection of all recognized homologs (para- and orthologs) under the label superfamily. These definitions result in groupings similar to the SCOP database, where a gene family is defined by sequence identity of 30% and greater, and superfamilies as "families whose proteins have low sequence identities but whose structures and, in many cases, functional features suggest that a common evolutionary origin is probable" [22].

Throughout the manuscript we use the term "gene family" to denote a collection of orthologs, where each species contributes one or in case of in-paralogs several genes to the family. Superfamilies are composed of families related to each other via significant BLAST hits, i.e., superfamilies correspond to single-linkage clusters. As a consequence, some superfamilies will contain families of orthologs that are joined via a single fusion protein. The implemented phylogenetic reconstruction of such a superfamily places the families of orthologs in distinct branches of the superfamily tree. See the section on phylogenetic reconstruction for further discussion and for alternatives.

The BranchClust algorithm is not restricted to the method of assembling superfamilies proposed in this article, and implemented in the BranchClust scripts. Rather, BranchClust allows one to analyze superfamilies assembled under any other selection criteria; e.g., the pre-computed families from COG or HOBCAGEN could be submitted for further BranchClust clustering.

### Technical limitations of BranchClust

Technical limitations of the BranchClust approach mostly are due to the exhaustive BLAST all-to-all searches and to the alignment of huge superfamilies rather than due to reconstruction and parsing of phylogenetic trees. Processing of 1500 superfamily trees for 13 taxa with BranchClust took about an hour on the University of Connecticut's biocluster (PowerPC G5 2.3 GHz, 2GB RAM), while the BLAST searches needed to assemble the superfamilies took about 12 hours with another day required for alignment of the superfamilies. All-against-all BLAST searches are needed only if the species in question are so diverged that direct BLAST comparisons between two distant species fails identify homologs that can be identified via intermediate species. Huge time savings result when only one-to-all BLAST searches are performed using only genes from the biggest genome from the dataset as query. However, these savings in computational time come at the price of inconsistency, because the way the superfamilies are selected, depends on the genome used as query genome. Using this shortcut, we tested BranchClust on a set of 319 bacterial and archaeal species available on the NCBI web-site as of April 2006. We took the genome of *Escherichia coli K12 *as starting genome and performed BLAST searches on a database composed of all 319 archaeal and bacterial species. Clustalw was able to align and reconstruct trees for superfamilies with up to 3000 genes (see the example of the ATP synthases superfamily for 319 taxa [see Additional file 3] and the BranchClust web-site [19]). Using the described one genome against all approach to form superfamilies, the total computation time from BLAST through to BranchClust selection for 319 species was less than 1 week.

If a diverse group of organisms is analyzed (e.g., all bacteria) and the MANY/FEW parameter is set too high, then orthologs that are present in only a small subgroup of the genomes (e.g. photosynthetic genes that are only present in photosynthetic bacteria) will be classified as outparalogs. Future improvement of the BranchClust algorithm will focus on training the algorithm to make context dependent choices for the MANY/FEW parameter. In case genomes from divergent organisms are analyzed we recommend to choose a smaller value for the MANY/FEW parameter, and to include superfamilies in the analyses that have representation in only a fraction of the analyzed genomes (see the BranchClust Tutorial available at [19]).

If genomes from divergent organisms are analyzed, the distinction between orthologs and paralogs might be difficult for proteins that experience high substitution rates. Especially in case of out-of-cluster-paralogs that are not separated from a cluster of orthologous genes by a long branch (e.g., sequence 15600541 in Figure 7), further analyses will be needed to test, if the sequence in question might be a misidentified member of a family of orthologs.

### Phylogenetic reconstruction

If only genomes from closely related organism are analyzed, the sequence divergence between orthologs will be much smaller than the divergence between ancient paralogs, and therefore, the overall result will not critically depend on the method for phylogenetic reconstruction chosen to calculate the phylogeny for the superfamily. The trees calculated to cluster the superfamily should not be considered substitutes for a more detailed phylogenetic assessment. Especially, if ancient paralogs are included in the superfamily, the relationship between the clusters of orthologous genes, and their relationship to other outparalogs remains uncertain. If these relations are to be determined more accurately, analyses needs to focus on the most conserved portions of the protein, whereas for within cluster relationships the use of the nucleotide sequences might be necessary, because synonymous substitutions might represent the only variability observed between sequences. A crucial determinant for the success of BranchClust is the difference in divergence between orthologs and divergence between outparalogs. Clustalw was selected for its speed, and because the generated trees having reasonable resolution at different levels of relatedness. By default we do not exclude poorly aligned parts from the analyses because for closer related sequences these poorly aligned stretches are the most informative. The chosen default approach biases the final tree topology towards the tree used during the progressive alignment [23]; however, this drawback is the price that is paid for using a single alignment for different levels of phylogenetic relationship.

We do not filter the initial alignments for the lengths of match as percent of the sequence length. Nevertheless, in none of the analyzed test cases did we find clusters of putative orthologs that were contaminated by fusion proteins. This is a testament to the robustness of the chosen tree building algorithm. Omitting a filter for minimum alignment lengths allowed inclusion of divergent sequences within one superfamily. If two families of orthologs are joined into a single superfamily through a fusion protein, clustalw places the families of orthologs on separate branches and BranchClust breaks them apart appropriately. If another tree reconstruction method is used, or if gaps are excluded globally, the superfamilies that are created through single fusion proteins need to be broken apart before BranchClust can be applied. One possibility is to only consider those BLAST hits in the formation of the superfamiles where the matches extend to more than 50% of the sequence lengths of both query and target sequence. Another solution is to shatter large superfamilies generated in single linkage clustering through application of Markov clustering [24,25]. This hybrid clustering approach was used successfully in [26] to avoid non-specific clusters resulting from matches to promiscuous domains.

If BranchClust is applied to genomes of a single genus or family, additional stringency could be applied in selecting members of a superfamily, and this in turn would allow excluding poorly aligned positions before further analysis. Even though the phylogenetic analysis used to cluster the superfamily is not very sophisticated, the utilization of a multiple sequence alignment and its analysis using a phylogenetic framework results in a powerful improvement with respect to the automated assembly of orthologs.

### Within cluster phylogeny

For the task of assembling gene families for a set of *n *taxa, we do not analyze the phylogeny within each branch, and thus no reference species tree is used or required. The phylogeny of the superfamily tree only is used to distinguish paralogs from clusters of orthologs. The crucial part of the supertree phylogeny is the branch that connects a cluster of orthologs to the outparalogs. A cluster of orthologs is recognized by virtue that many of the included genomes are represented in this cluster (Figure 4). This independence from a reference species phylogeny often will be an advantage. In case of many closely related genomes, too few substitution events have occurred to reconstruct the genomes' relationships from a single gene. In case of prokaryotes single genes and operons can be transferred between species [27,28] and many other processes and artifacts are known to yield differences between individual gene phylogeny and the consensus phylogeny [29]. However, additional phylogenetic analysis is needed to compare the evolutionary history between paralogous families. The topological congruence of paralogous branches could be used to infer the root of the superfamily tree (see results for details and Figure 6), allowing to trace back the evolutionary history of molecules to times before the organismal common ancestor came into existence [30-33].

BranchClust does not alleviate the need to compare individual gene families to the consensus or species phylogenies – rather it provides for an assembly of families of putative orthologs that is a prerequisite for studying their evolution. Mapping the sequence of duplication events onto a species tree, and finding instances of orthologous replacement are important to understand the evolution of a superfamily. Knowledge of the species phylogeny is required to interpret the data. For example, the case of the ATP synthase superfamily for 30 bacteria and archaea ([see Additional file 2] and examples discussed at the BranchClust web-site [19]) a sequence from the archaeon *Methanosarcina *groups within bacterial sequences for both ATP-A and ATP-B genes, and two bacterial homologs, from *Deinococcus *and *Thermus*, are located within a group of archaeal homologs also for both ATP-A and ATP-B genes [34-37]. BranchClust does not perform the analyses necessary to identify these events, but it provides a greatly improved, automated tool to assemble the starting materials on which to do these studies.

### The universal genome core

One surprising outcome of our analysis was the large number of families that were well represented in both the archaeal and bacterial domains of life (see Table 1). The strict core, i.e. genes that are found in all organisms remains small, and is expected to shrink further as more genome sequences become available [12]. The reason for this trend is that specialized organisms, in particular parasites and symbionts, often have reduced genomes, sometimes lacking even important functions like ATP synthesis and chemiosmotic coupling [38]. However, even with only slightly relaxed conditions, the number of gene families present in most archaeal and bacterial genomes was surprisingly high. This finding re-affirms the shared ancestry of all life and is testament to the conservative nature of evolution: re-use of something already invented appears to be preferred over re-invention from scratch. The number of gene families with near universal distribution among prokaryotes appears to be higher than previously determined, possibly because in previous analyses the selection of orthologs was so stringent that many legitimate orthologs were excluded.

## Conclusion

The RBH method frequently fails in assembling families of orthologous genes because this method fails to handle paralogs, in particular inparalogs, a finding acknowledged in many reports using automated assembly of orthologous sets (e.g., [7,39]).

BranchClust provides an automated, robust approach to assemble families of orthologs. It effectively selects complete and incomplete clusters of putatively orthologous genes, including inparalogs arising through lineage specific gene amplification. The use of BranchClust will allow one to include larger portions of completely sequenced genomes into genome based phylogenetic reconstructions, thereby allowing phylogenomics to move away from the currently dominating reconstruction of trees describing the history of the transcription and translation machinery [40] to analyses that include metabolic pathways that might be present only in a subset of the analyzed genomes.

## Methods

### List of taxa used in analysis

#### 4 taxa: 2 bacteria and 2 archaea

##### 2 bacteria

*Escherichia coli*, *Bacillus subtilis*

##### 2 archaea

*Methanosarcina mazei*, *Sulfolobus solfataricus*.

#### 13 gamma proteobacteria

*Buchnera aphidicola*, *Escherichia coli*, *Haemophilus influenzae*, *Pasteurella multocida*, *Pseudomonas aeruginosa*, *Salmonella typhimurium*, *Vibrio cholerae*, *Wigglesworthia glossinidia*, *Xanthomonas campestris*, *Xanthomonas axonopodis*, *Xylella fastidiosa*, *Yersinia pestis *KIM, *Yersinia pestis *CO92.

#### 30 taxa: 16 bacteria and 14 archaea

##### 16 bacteria

*Aquifex aeolicus, Bacillus subtilis, Chlorobium tepidum, Corynebacterium glutamicum, Deinococcus radiodurans, Gobacillus kaustophilus, Geobacter sulfurreducens, Gloeobacter violaceus, Nostoc sp., Pseudomonas aeruginosa, Rhodopirellula baltica, Rhodopseudomonas palustris, Streptococcus thermophilus, Streptomyces coelicolor, Thermotoga maritime, Thermus thermophilus*.

##### 14 archaea

*Aeropyrum pernix, Archaeoglobus fulgidus, Haloarcula marismortui, Halobacterium sp., Methanococcus maripaludis, Methanopyrus kandleri, Methanosarcina acetivorans, Methanothermobacter thermautotrophicus, Nanoarchaeum equitans, Pyrobaculum aerophilum, Pyrococcus abyssi, Sulfolobus solfataricus, Thermococcus kodakaraensis, Thermoplasma acidophilum*.

**319 taxa**: the list can be found at [19].

### Sequence alignment and tree building

Currently, BranchClust by default uses clustalw 1.83 [41] for both sequence alignment and tree reconstruction using a distance method with Kimura correction. Other programs that are fast and use a command line interface can easily be substituted: e.g. MUSCLE [42] for sequence alignment, phyml [43], TREEPUZZLE [44] in conjunction with neighbor joining [45]) for phylogenetic tree reconstruction.

### BranchClust analysis

Please see the BranchClust tutorial at [19] for guidance on all procedures of the method from downloading complete genomes to applying the BranchClust algorithm.

For the analysis of 4, 13 and 30 taxa we performed exhaustive all-to-all BLAST searches to select superfamilies. For the case of 319 taxa we tested one-to-all BLAST approach choosing the genome of *Escherichia coli K12 *as starting genome. The BranchClust method was applied with the following MANY/FEW parameters: MANY/FEW = 3 for 4 taxa, MANY/FEW = 8 for 13 gamma proteobacteria, MANY/FEW = 24 for 30 taxa, and MANY/FEW = 150 for 319 taxa.

## Availability and requirements

**Project name**: BranchClust

**Project home page**: 

**Operating system(s)**: UNIX, MAC OS X

**Programming language**: Perl

**Licence**: GNU General Public License

**Any restrictions to use by non-academics**: Contact authors

## Authors' contributions

MP and JPG collaborated in the developing of the algorithm. MP implemented the algorithm in the perl-script, evaluated method for different sets of species and created and maintains the BranchClust web-site. MP and JPG collaborated in writing the manuscript. Both authors read and approved the final manuscript.

## Supplementary Material

Additional file 1 ATP synthase superfamily for 13 gamma proteobacteria. Superfamily of ATP synthases alpha and beta subunits, flagella and type III secretion system ATPases, and Rho-termination factors. The superfamily was assembled by all-to-all BLAST searches; BranchClust was applied with MANY/FEW = 8. Click here for file

Additional file 2 ATP synthase superfamily for 30 bacteria and archaea. Superfamily of ATP synthases alpha and beta subunits, flagella and type III secretion system ATPases, and Rho-termination factors. The superfamily was assembled by all-to-all BLAST searches; BranchClust was applied with MANY/FEW = 10. Click here for file

Additional file 3 ATP synthase superfamily for 317 bacteria and archaea. Superfamily of ATP synthases alpha and beta subunits, flagella and type III secretion system ATPases, and Rho-termination factors. The superfamily was assembled by on-to-all BLAST searches using genome of *Escherichia coli K12 *as a starting genome; BranchClust was applied with MANY/FEW = 150. Click here for file

Additional file 4 Superfamily of cell division proteins, ribonucleases E, ATP synthase chain B and hypothetical proteins for 13 gamma proteobacteria. The superfamily was assembled by all-to-all BLAST searches; BranchClust was applied with MANY/FEW = 8. Click here for file

Additional file 5 Superfamily DNA topoisomerase IV subunit B and DNA gyrase subunit IV for 13 gamma proteobacteria. The superfamily was assembled by all-to-all BLAST searches; BranchClust was applied with MANY/FEW = 8. Click here for file

Additional file 6 Superfamily of signal recognition particle proteins, chromosomal replication initiators, insertion sequence protein for 13 gamma proteobacteria. The superfamily was assembled by all-to-all BLAST searches; BranchClust was applied with MANY/FEW = 8. Click here for file

Additional file 7 Superfamily of elongation factors, peptide chain release factors and GTP-binding proteins for 13 gamma proteobacteria. The superfamily was assembled by all-to-all BLAST searches; BranchClust was applied with MANY/FEW = 8. Click here for file

Additional file 8 Superfamily of methyltransferases for 13 gamma proteobacteria. The superfamily was assembled by all-to-all BLAST searches; BranchClust was applied with MANY/FEW = 8. Click here for file

Additional file 9 Superfamily of universal stress protein and amino-acid transporters for 14 archaea. The superfamily was assembled by all-to-all BLAST searches; BranchClust was applied with MANY/FEW = 8. Click here for file

Additional file 10 Superfamily of kinases for 14 archaea. The superfamily was assembled by all-to-all BLAST searches; BranchClust was applied with MANY/FEW = 8. Click here for file

Additional file 11 Superfamily of aminotransferases for 14 archaea. The superfamily was assembled by all-to-all BLAST searches; BranchClust was applied with MANY/FEW = 8. Click here for file

Additional file 12 Superfamily of potential transcriptional regulators for 14 archaea. The superfamily was assembled by all-to-all BLAST searches; BranchClust was applied with MANY/FEW = 8. Click here for file

Additional file 13 Superfamily of potential transcriptional regulators for 14 archaea. The superfamily was assembled by all-to-all BLAST searches; BranchClust was applied with MANY/FEW = 8. Click here for file

Additional file 14 Superfamily of reductases, dehydrogenases, NADH oxidases for 16 bacteria and 14 archaea. The superfamily was assembled by all-to-all BLAST searches; BranchClust was applied with MANY/FEW = 24. Click here for file

Additional file 15 Superfamily of translation elongation factors, peptide chain release factors and GTP-binding proteins for 16 bacteria and 14 archaea. The superfamily was assembled by all-to-all BLAST searches; BranchClust was applied with MANY/FEW = 24. Click here for file

Additional file 16 Superfamily of replication factors C and DNA polymerases III for 16 bacteria and 14 archaea. The superfamily was assembled by all-to-all BLAST searches; BranchClust was applied with MANY/FEW = 24. Click here for file

Additional file 17 Superfamily of ATP dependent RNA and DNA helicases for 16 bacteria and 14 archaea. The superfamily was assembled by all-to-all BLAST searches; BranchClust was applied with MANY/FEW = 24. Click here for file

Additional file 18 Superfamily of ABC transporters for 16 bacteria and 14 archaea. The superfamily was assembled by all-to-all BLAST searches; BranchClust was applied with MANY/FEW = 24. Click here for file
