# Supplementary figures and images for: BranchClust: a phylogenetic algorithm for selecting gene families
Source: BMC Bioinformatics. 2007 Apr 10;8:120. doi: 10.1186/1471-2105-8-120 (PMC1853112; doi:10.1186/1471-2105-8-120)

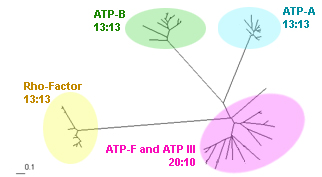

Supplement: Additional file 1 — ATP synthase superfamily for 13 gamma proteobacteria. Superfamily of ATP synthases alpha and beta subunits, flagella and type III secretion system ATPases, and Rho-termination factors. The superfamily was assembled by all-to-all BLAST searches; BranchClust was applied with MANY/FEW = 8. [file 1471-2105-8-120-S1.jpeg]

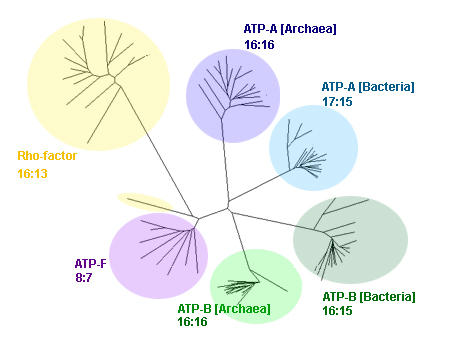

Supplement: Additional file 2 — ATP synthase superfamily for 30 bacteria and archaea. Superfamily of ATP synthases alpha and beta subunits, flagella and type III secretion system ATPases, and Rho-termination factors. The superfamily was assembled by all-to-all BLAST searches; BranchClust was applied with MANY/FEW = 10. [file 1471-2105-8-120-S2.jpeg]

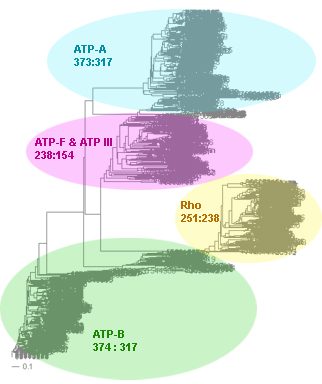

Supplement: Additional file 3 — ATP synthase superfamily for 317 bacteria and archaea. Superfamily of ATP synthases alpha and beta subunits, flagella and type III secretion system ATPases, and Rho-termination factors. The superfamily was assembled by on-to-all BLAST searches using genome of Escherichia coli K12 as a starting genome; BranchClust was applied with MANY/FEW = 150. [file 1471-2105-8-120-S3.jpeg]

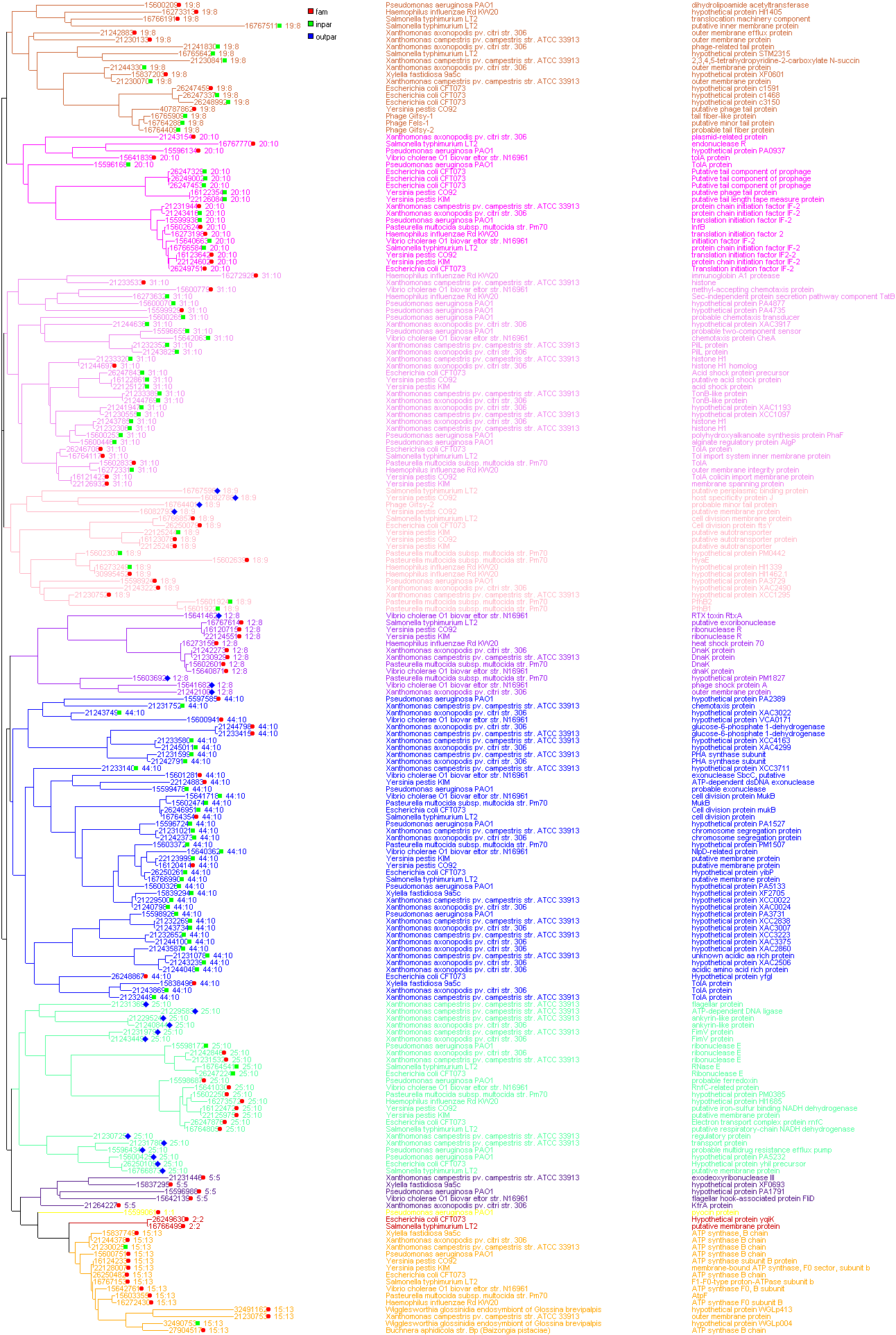

Supplement: Additional file 4 — Superfamily of cell division proteins, ribonucleases E, ATP synthase chain B and hypothetical proteins for 13 gamma proteobacteria. The superfamily was assembled by all-to-all BLAST searches; BranchClust was applied with MANY/FEW = 8. [file 1471-2105-8-120-S4.png]

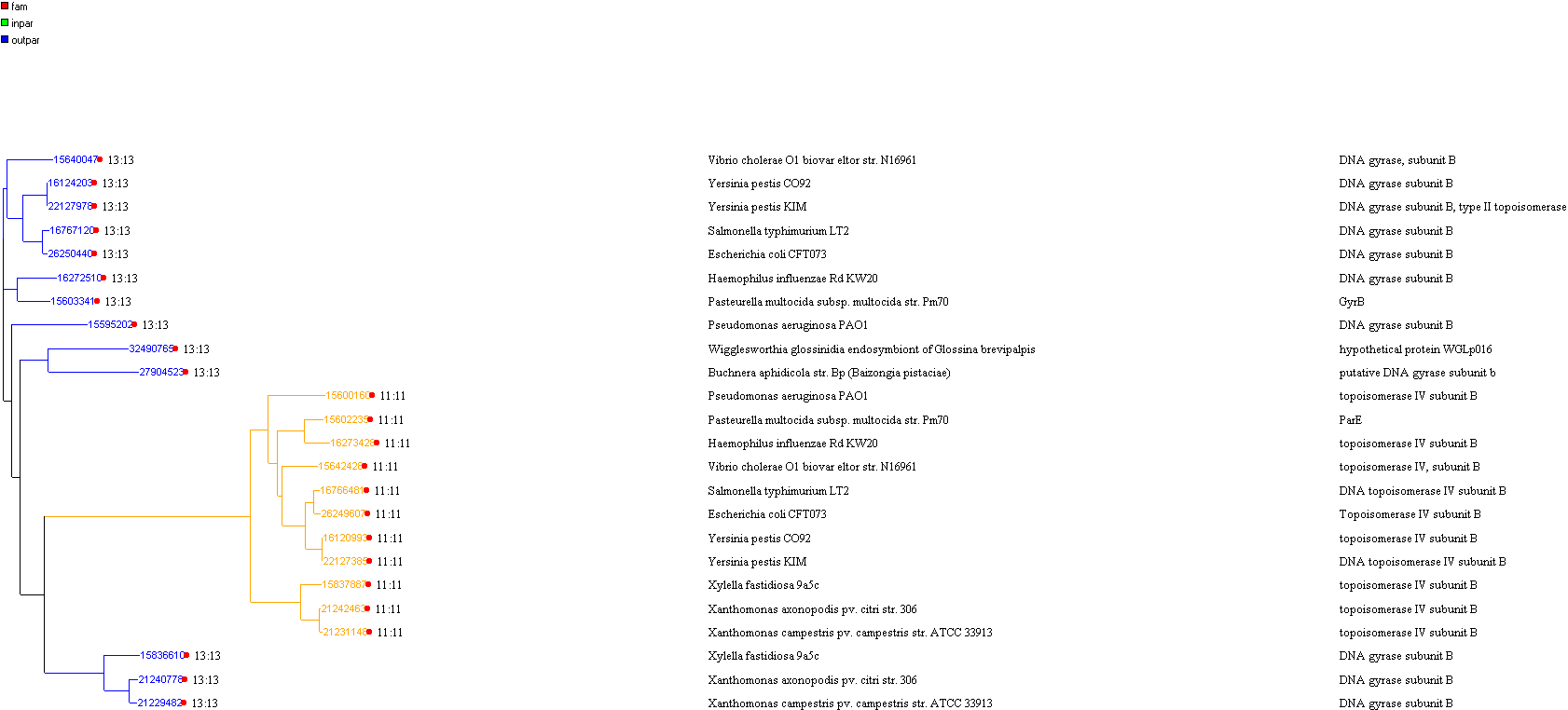

Supplement: Additional file 5 — Superfamily DNA topoisomerase IV subunit B and DNA gyrase subunit IV for 13 gamma proteobacteria. The superfamily was assembled by all-to-all BLAST searches; BranchClust was applied with MANY/FEW = 8. [file 1471-2105-8-120-S5.png]

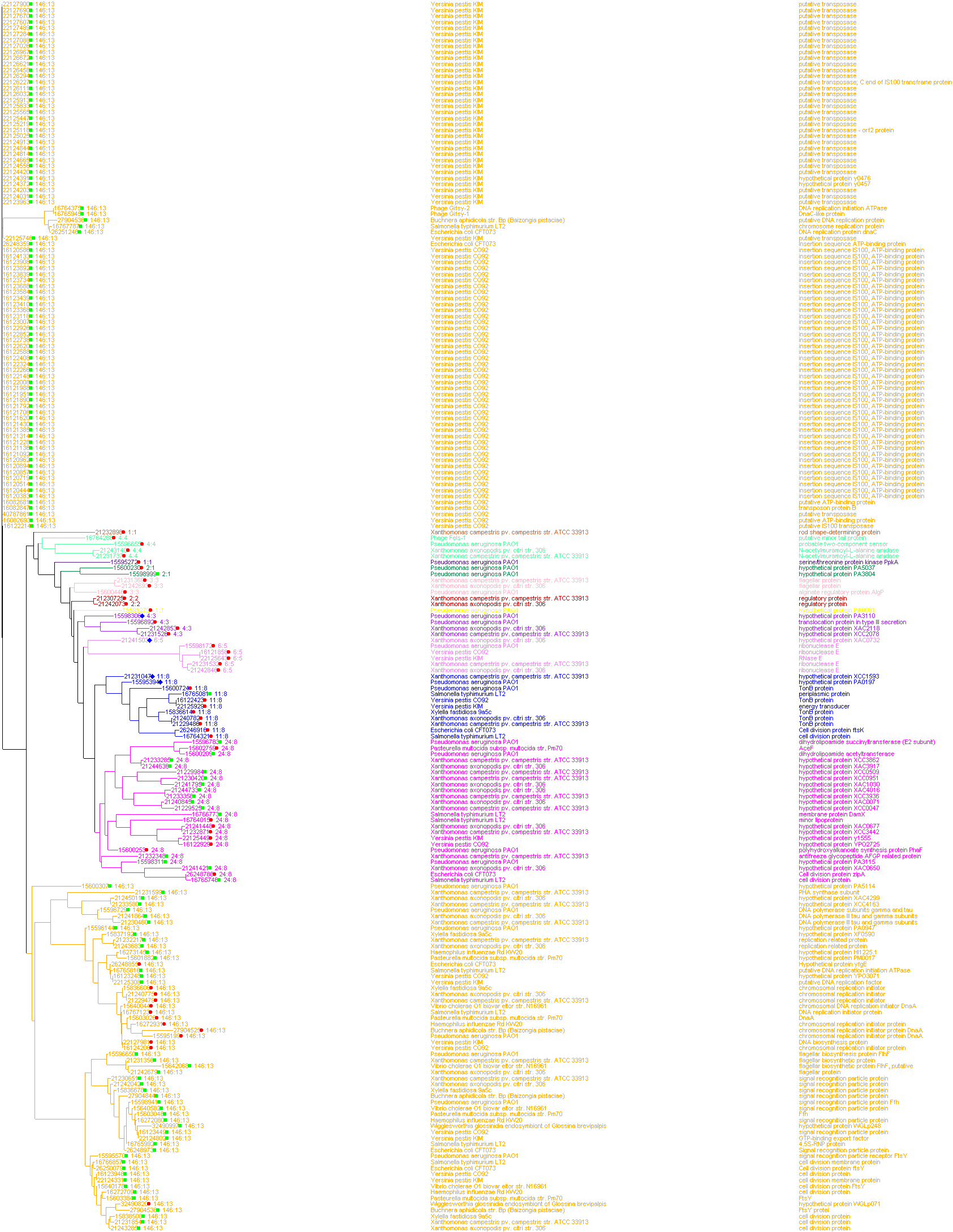

Supplement: Additional file 6 — Superfamily of signal recognition particle proteins, chromosomal replication initiators, insertion sequence protein for 13 gamma proteobacteria. The superfamily was assembled by all-to-all BLAST searches; BranchClust was applied with MANY/FEW = 8. [file 1471-2105-8-120-S6.png]

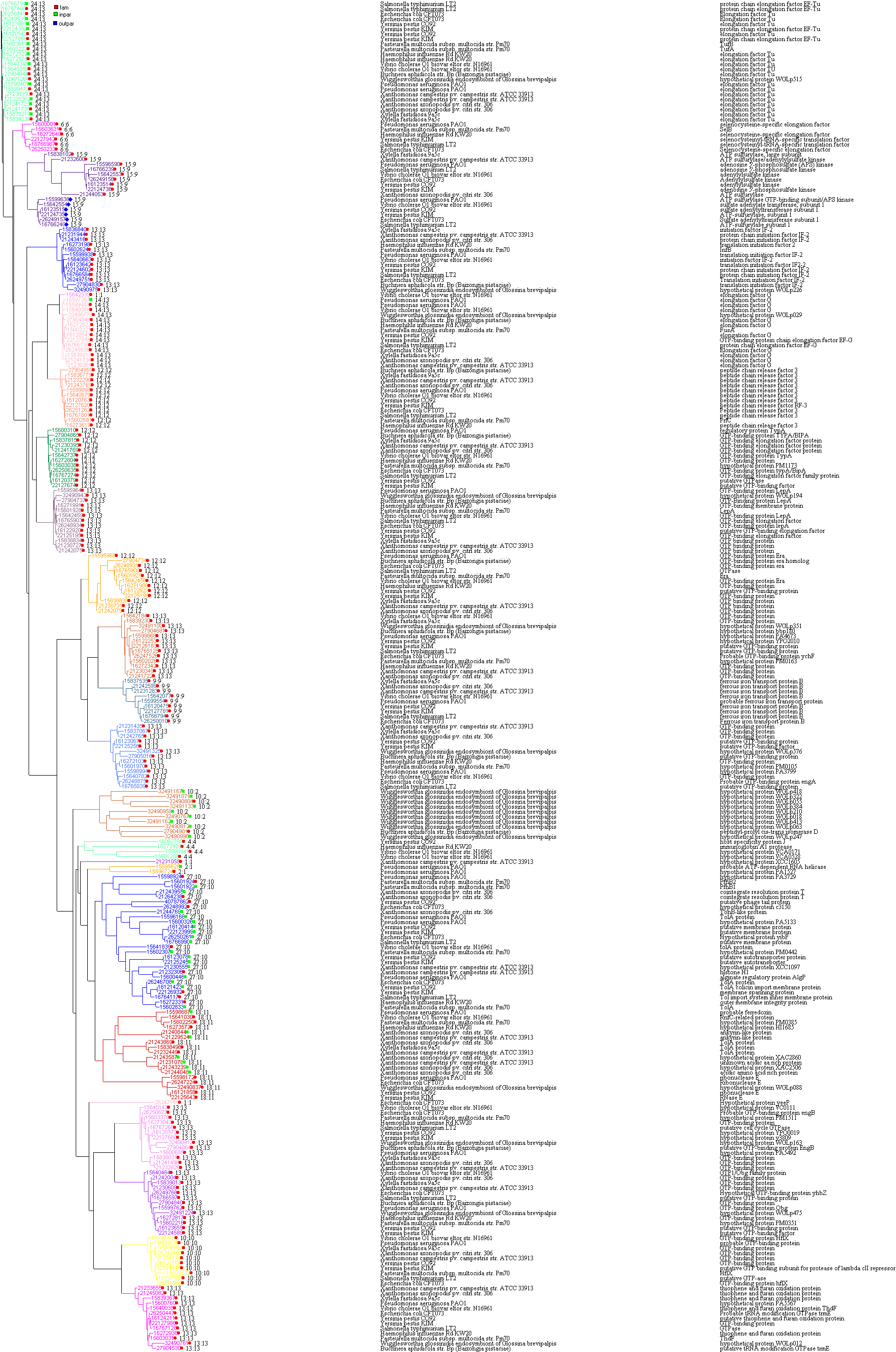

Supplement: Additional file 7 — Superfamily of elongation factors, peptide chain release factors and GTP-binding proteins for 13 gamma proteobacteria. The superfamily was assembled by all-to-all BLAST searches; BranchClust was applied with MANY/FEW = 8. [file 1471-2105-8-120-S7.png]

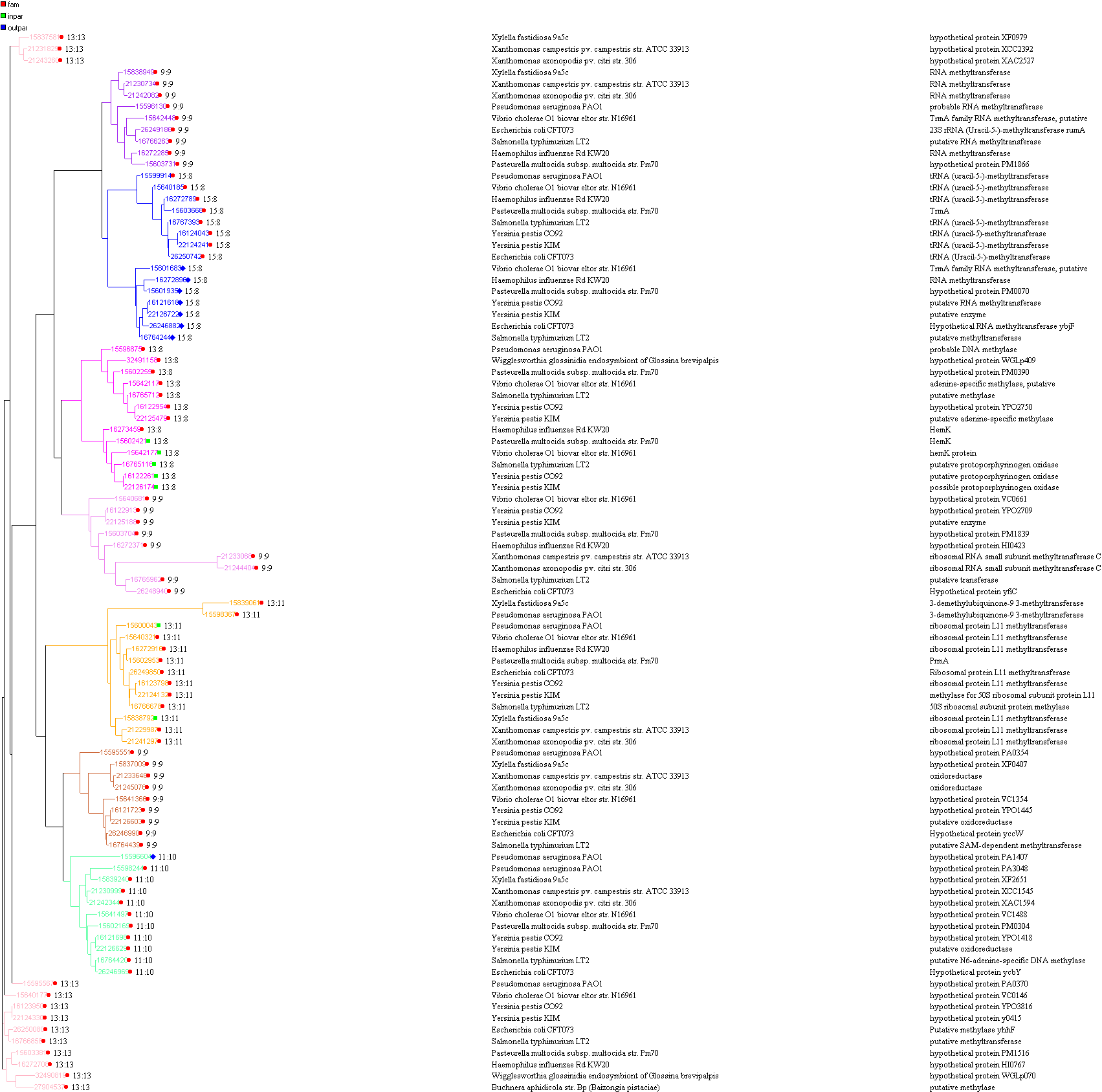

Supplement: Additional file 8 — Superfamily of methyltransferases for 13 gamma proteobacteria. The superfamily was assembled by all-to-all BLAST searches; BranchClust was applied with MANY/FEW = 8. [file 1471-2105-8-120-S8.png]

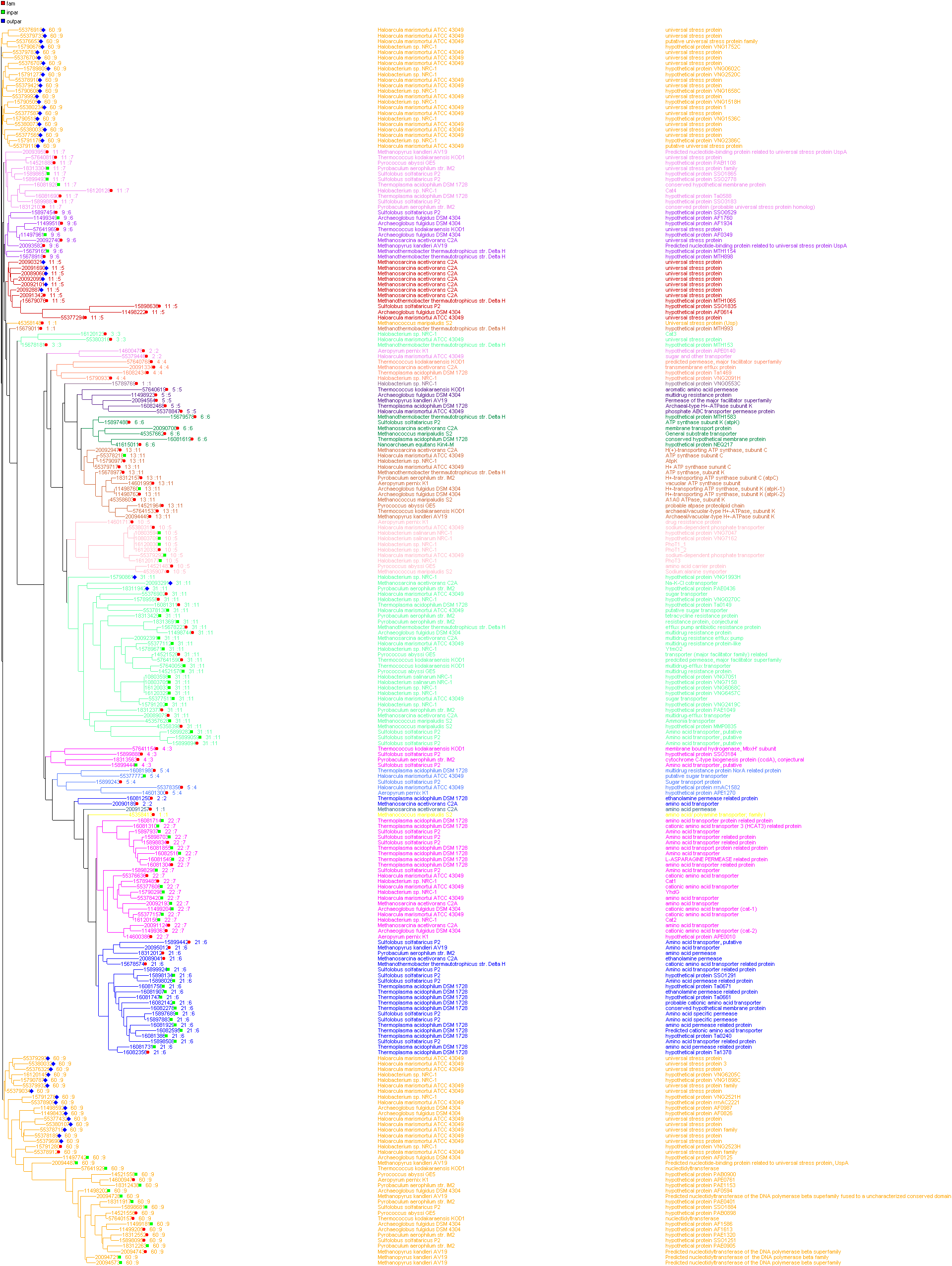

Supplement: Additional file 9 — Superfamily of universal stress protein and amino-acid transporters for 14 archaea. The superfamily was assembled by all-to-all BLAST searches; BranchClust was applied with MANY/FEW = 8. [file 1471-2105-8-120-S9.png]

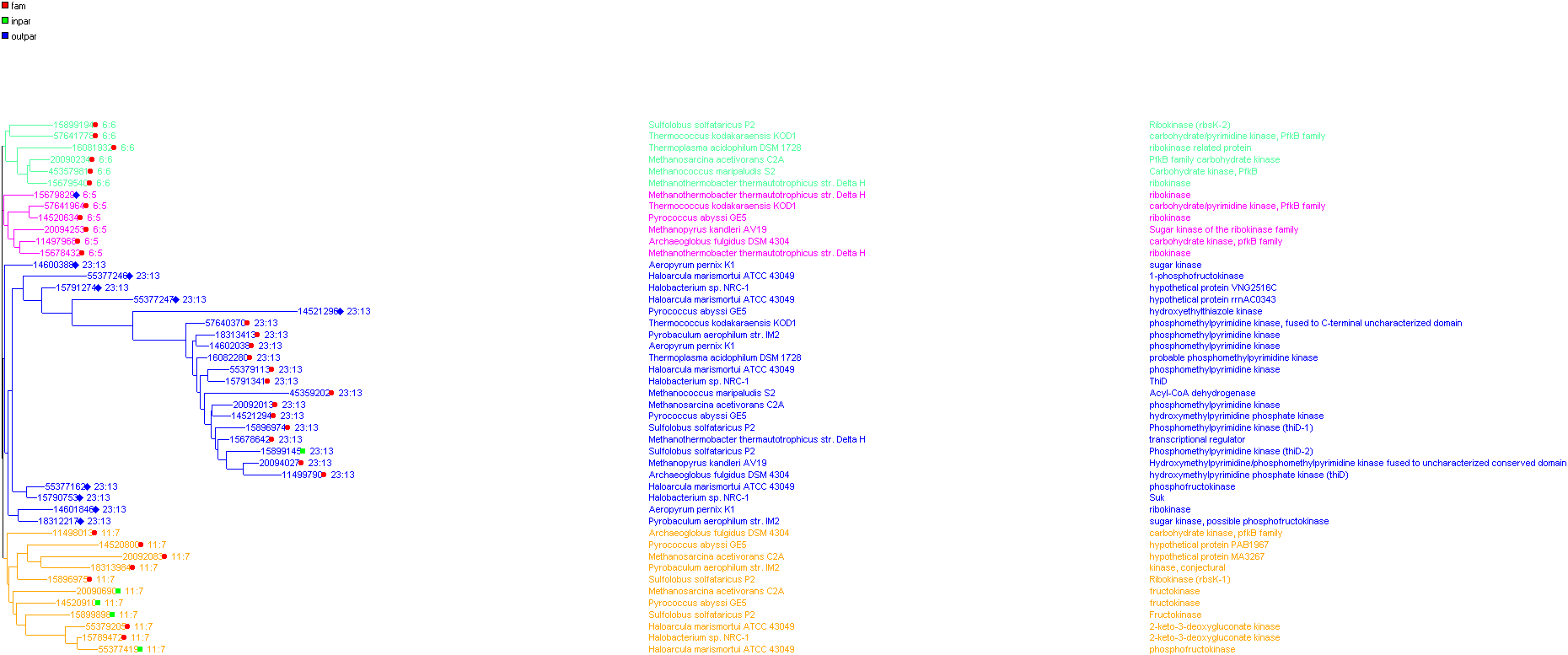

Supplement: Additional file 10 — Superfamily of kinases for 14 archaea. The superfamily was assembled by all-to-all BLAST searches; BranchClust was applied with MANY/FEW = 8. [file 1471-2105-8-120-S10.png]

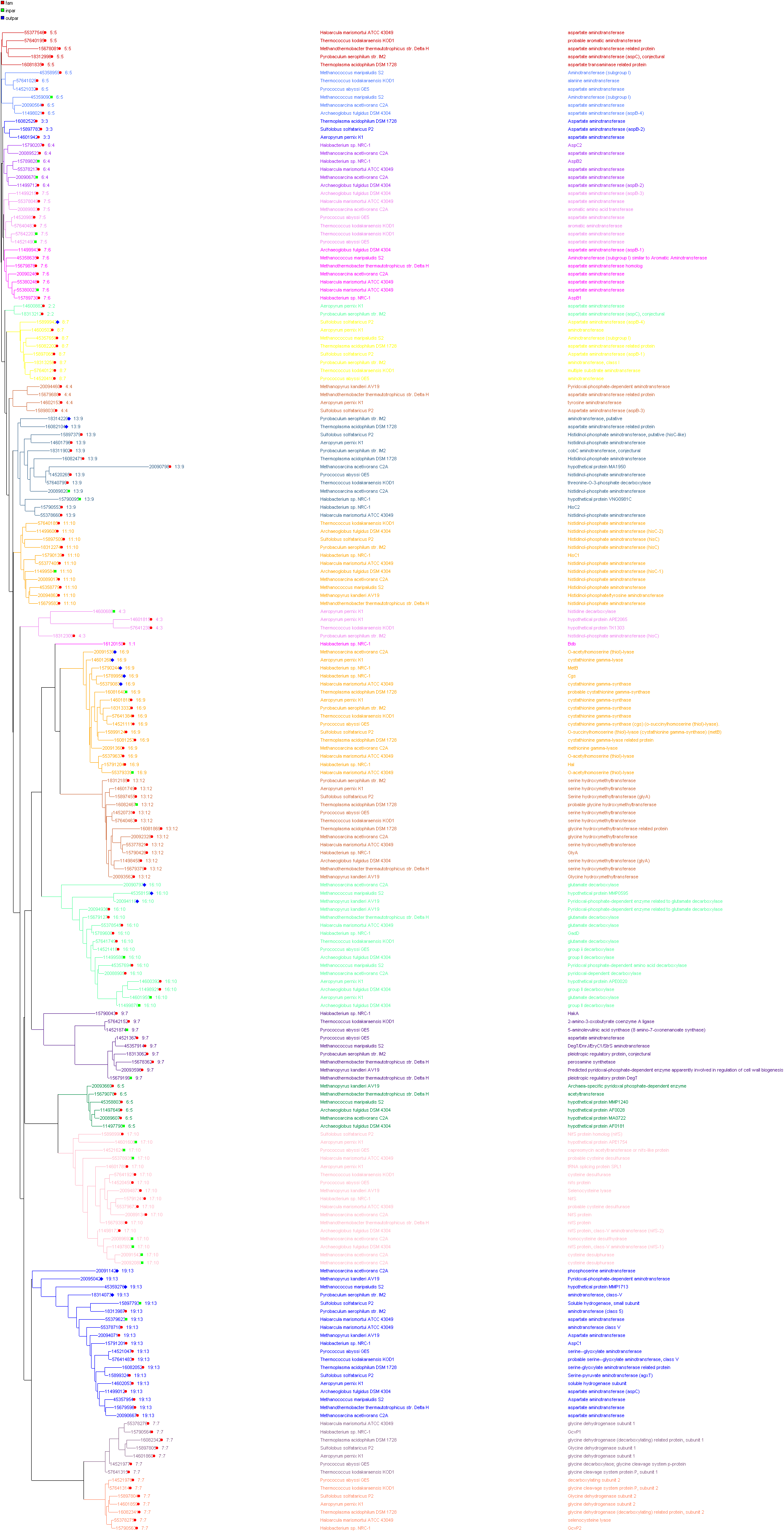

Supplement: Additional file 11 — Superfamily of aminotransferases for 14 archaea. The superfamily was assembled by all-to-all BLAST searches; BranchClust was applied with MANY/FEW = 8. [file 1471-2105-8-120-S11.png]

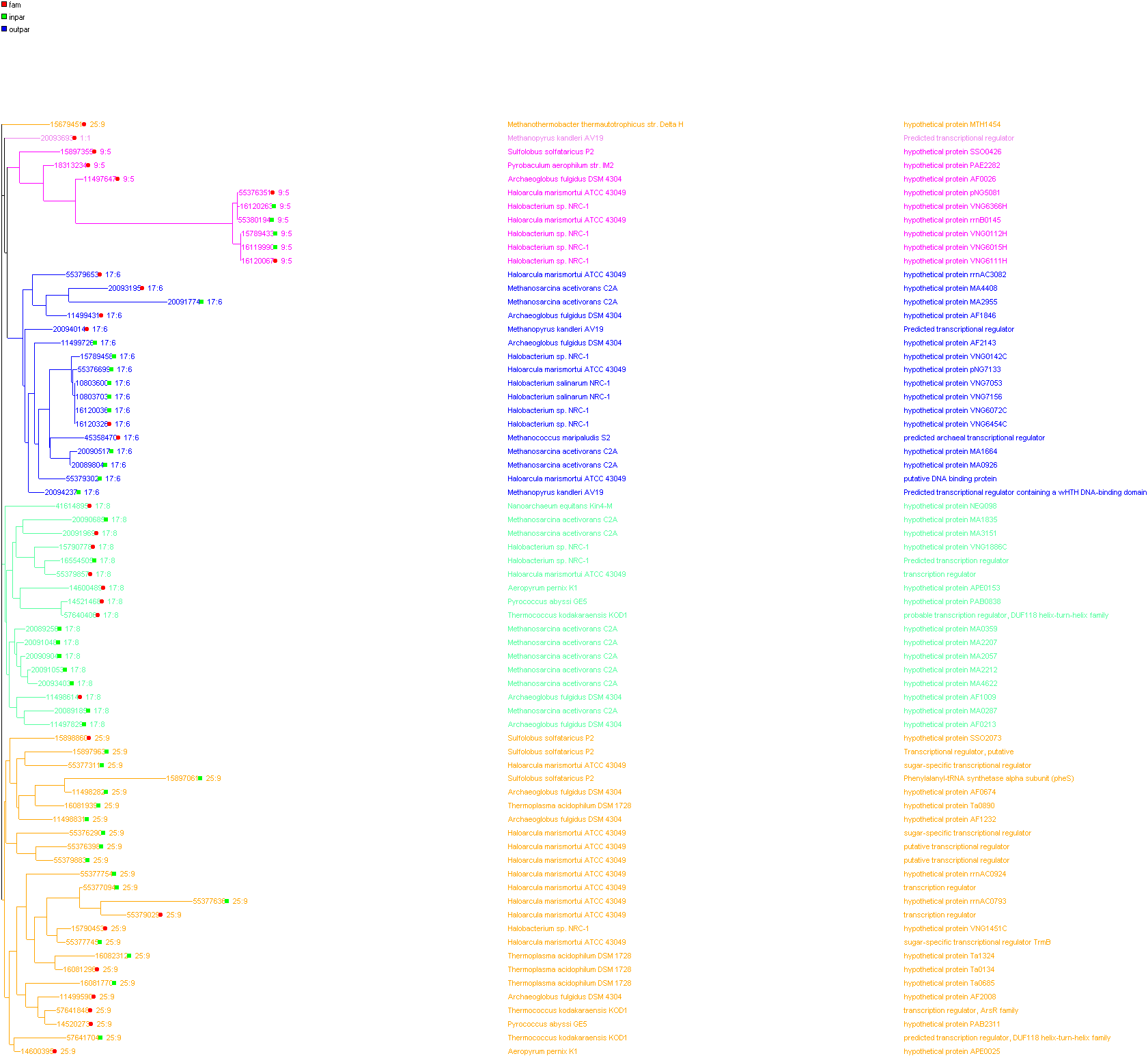

Supplement: Additional file 12 — Superfamily of potential transcriptional regulators for 14 archaea. The superfamily was assembled by all-to-all BLAST searches; BranchClust was applied with MANY/FEW = 8. [file 1471-2105-8-120-S12.png]

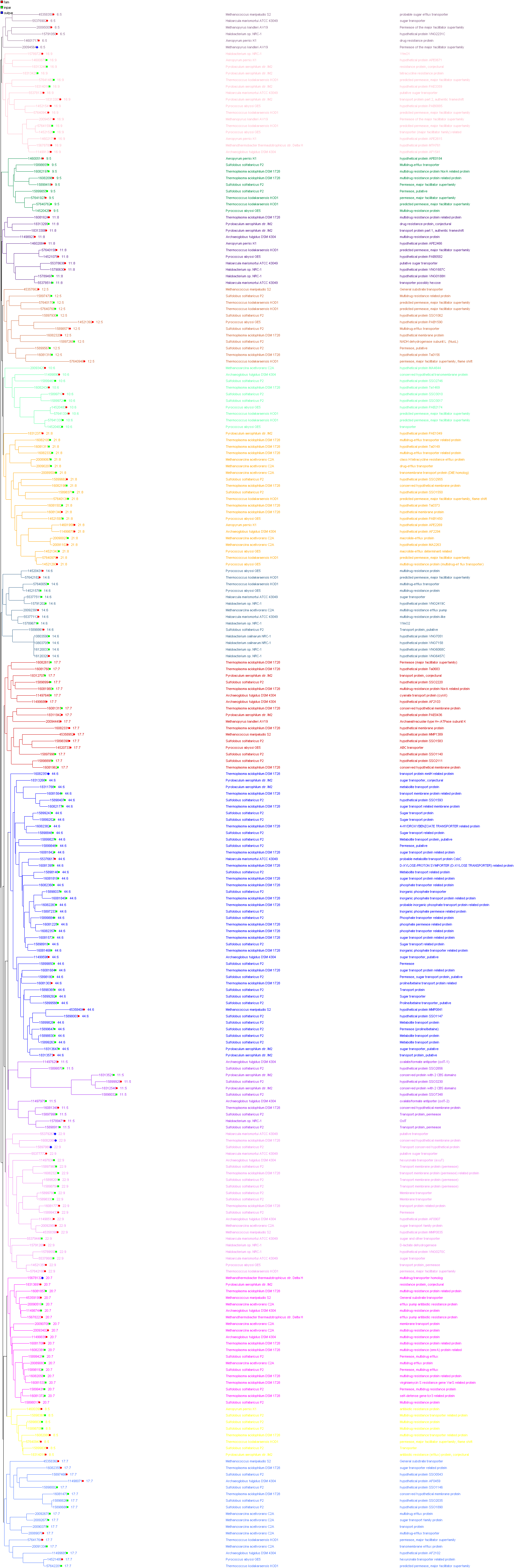

Supplement: Additional file 13 — Superfamily of potential transcriptional regulators for 14 archaea. The superfamily was assembled by all-to-all BLAST searches; BranchClust was applied with MANY/FEW = 8. [file 1471-2105-8-120-S13.png]

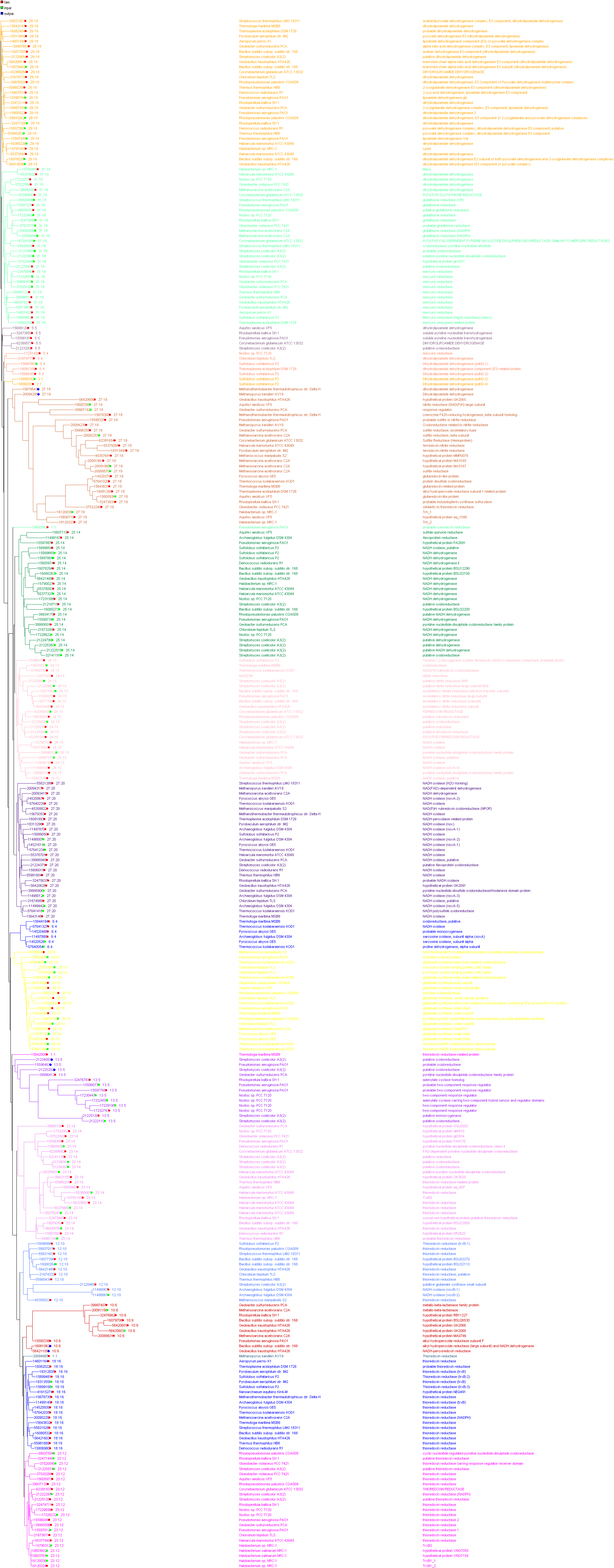

Supplement: Additional file 14 — Superfamily of reductases, dehydrogenases, NADH oxidases for 16 bacteria and 14 archaea. The superfamily was assembled by all-to-all BLAST searches; BranchClust was applied with MANY/FEW = 24. [file 1471-2105-8-120-S14.png]

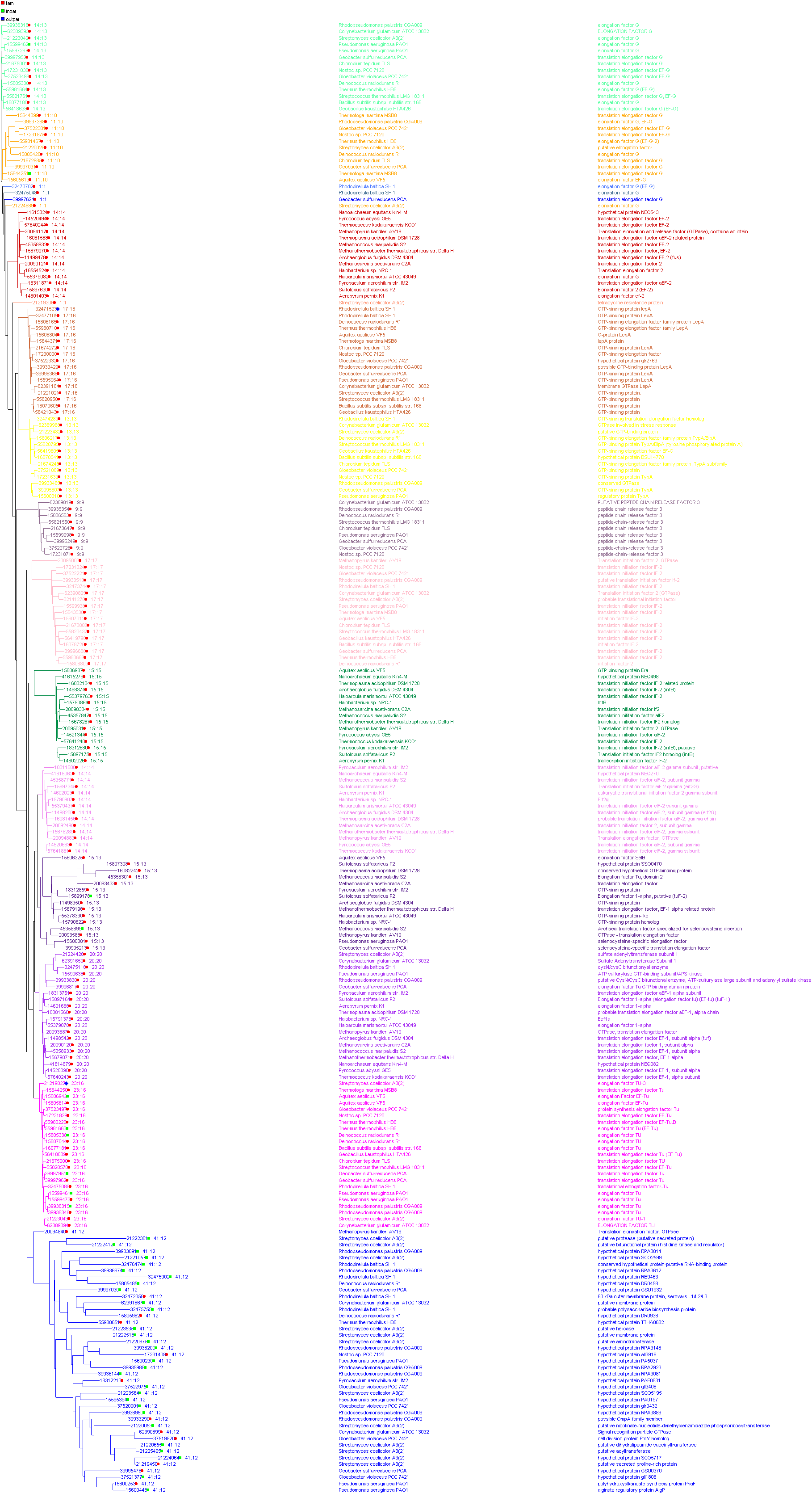

Supplement: Additional file 15 — Superfamily of translation elongation factors, peptide chain release factors and GTP-binding proteins for 16 bacteria and 14 archaea. The superfamily was assembled by all-to-all BLAST searches; BranchClust was applied with MANY/FEW = 24. [file 1471-2105-8-120-S15.png]

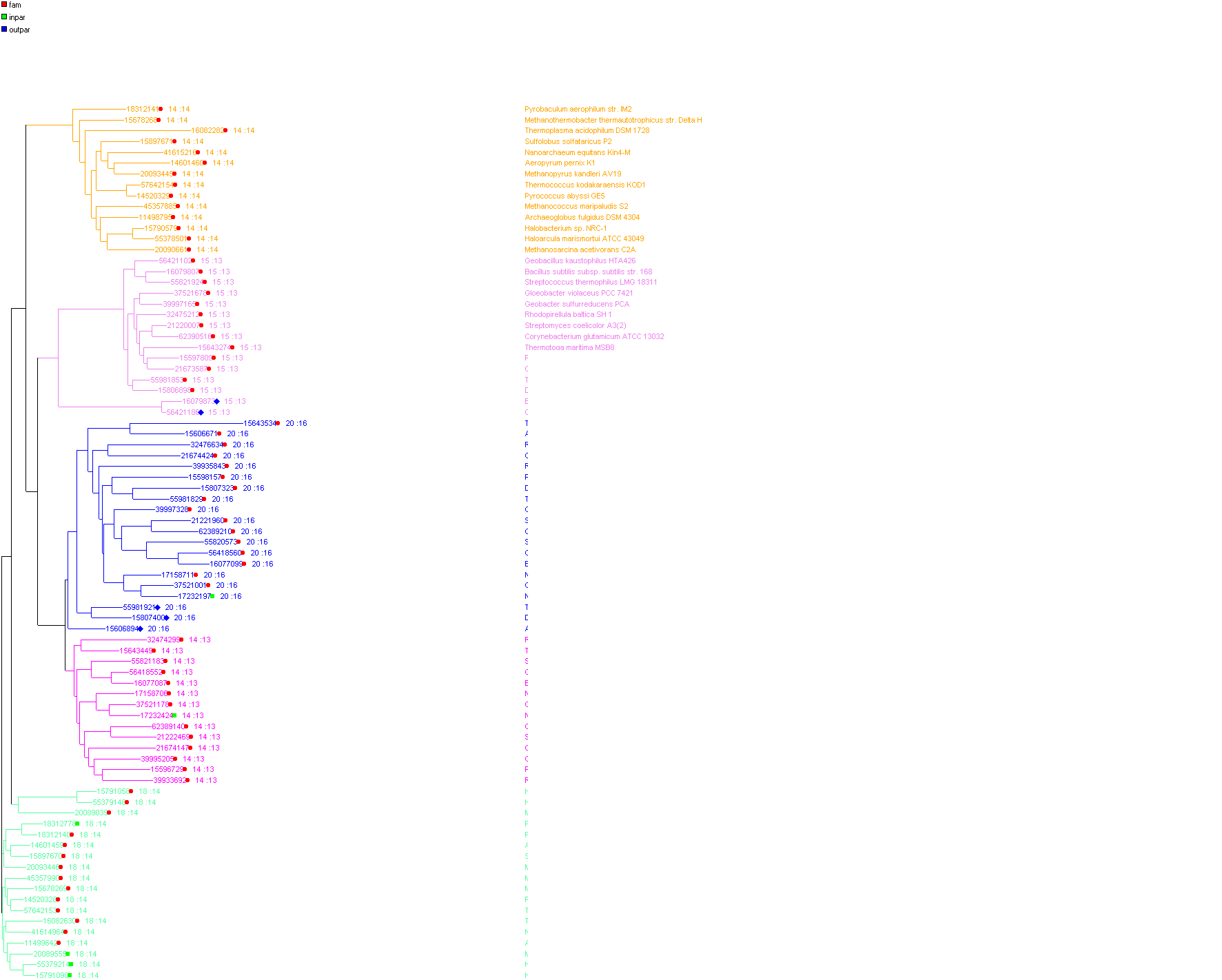

Supplement: Additional file 16 — Superfamily of replication factors C and DNA polymerases III for 16 bacteria and 14 archaea. The superfamily was assembled by all-to-all BLAST searches; BranchClust was applied with MANY/FEW = 24. [file 1471-2105-8-120-S16.png]

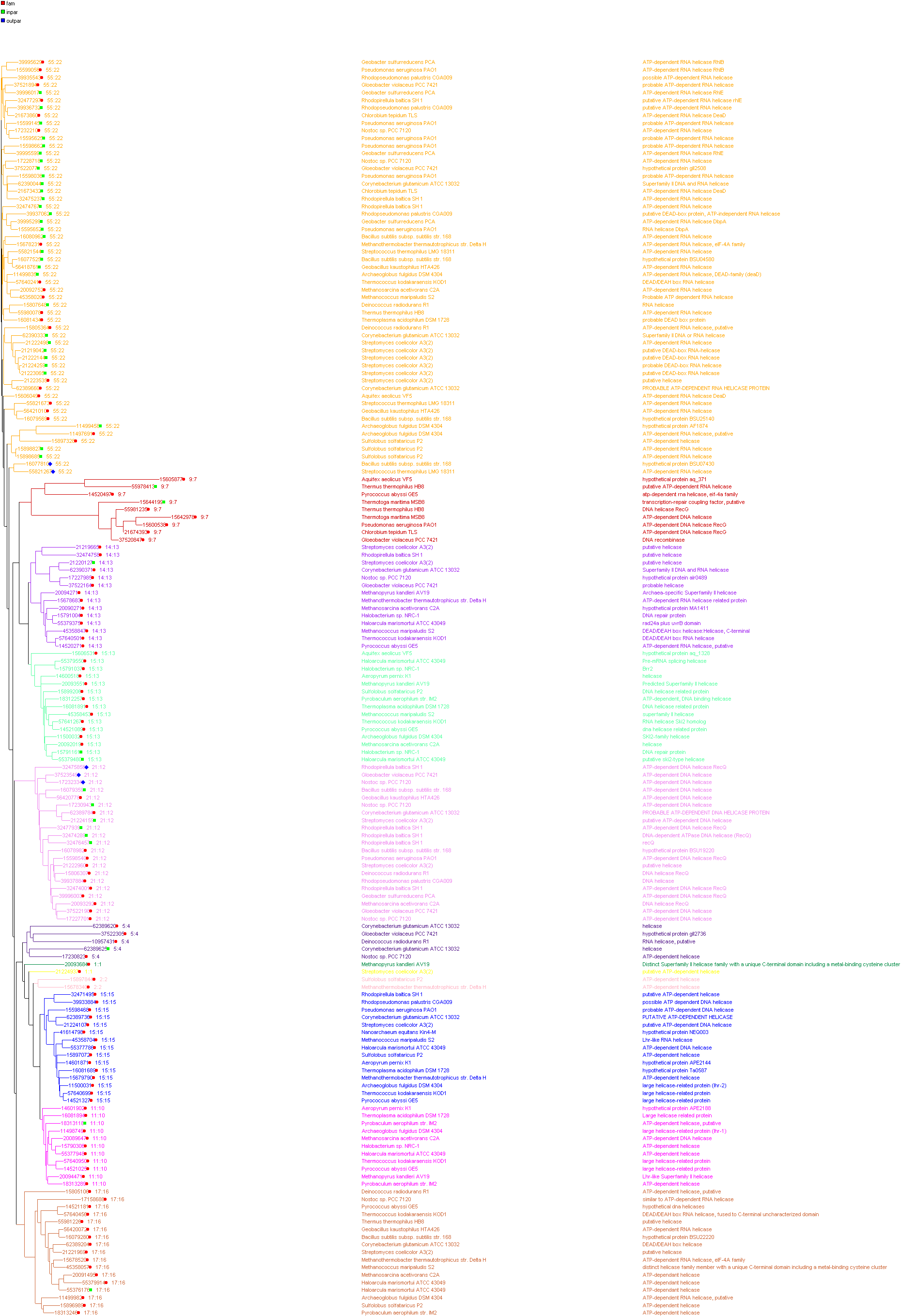

Supplement: Additional file 17 — Superfamily of ATP dependent RNA and DNA helicases for 16 bacteria and 14 archaea. The superfamily was assembled by all-to-all BLAST searches; BranchClust was applied with MANY/FEW = 24. [file 1471-2105-8-120-S17.png]

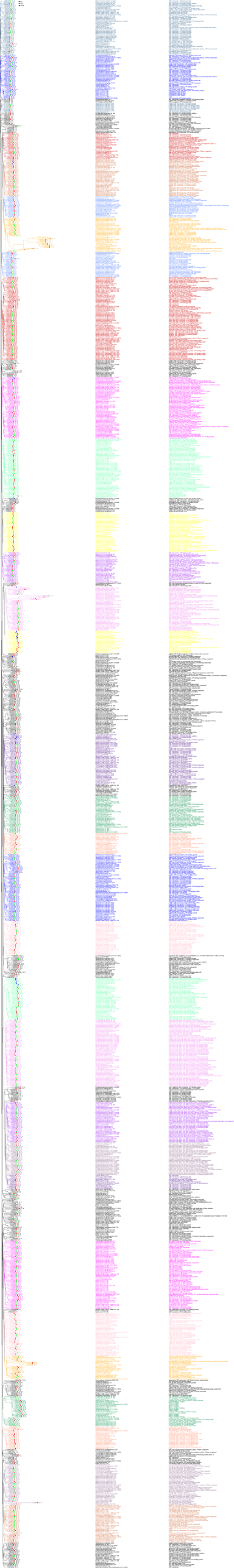

Supplement: Additional file 18 — Superfamily of ABC transporters for 16 bacteria and 14 archaea. The superfamily was assembled by all-to-all BLAST searches; BranchClust was applied with MANY/FEW = 24. [file 1471-2105-8-120-S18.png]
